# Efficacy and safety of stem cells in the treatment of glaucoma: systematic review and meta-analysis based on animal experiments

**DOI:** 10.3389/fphar.2025.1587440

**Published:** 2025-07-01

**Authors:** Danning Wu, Yanjing Liu, Xia Zhang, Ruixuan Zhang, Shaopeng Wang, Hui Lu, Tao Yue

**Affiliations:** ^1^ School of Clinical Medicine, Shandong Second Medical University, Weifang, Shandong, China; ^2^ Key Laboratory of Ophthalmology and Visual Science of Zibo City, Zibo Central Hospital, Shandong Second Medical University, Zibo, Shandong, China; ^3^ Department of Geriatrics, Zibo Central Hospital, Shandong Second Medical University, Zibo, Shandong, China

**Keywords:** stem cells, glaucoma, meta-analysis, randomized controlled trials, animal experiment

## Abstract

**Purpose:**

Glaucoma, characterized by progressive retinal ganglion cell (RGC) degeneration and optic nerve atrophy, remains a leading global cause of irreversible blindness, despite advancements in clinical management. While current therapies predominantly focus on lowering intraocular pressure (IOP), neuroprotective strategies to preserve visual function remain an unmet clinical need. Stem cells exhibit high proliferative capacity and multilineage differentiation potential, demonstrating notable efficacy in glaucoma treatment. Emerging preclinical evidence further highlights dual neuroprotective mechanisms (i.e., paracrine neurotrophic support and cellular replacement) of stem cell-based interventions. Accordingly, this systematic review and meta-analysis evaluated the therapeutic efficacy and safety of stem cell transplantation in experimental glaucoma models, with a focus on functional outcomes (IOP modulation), structural preservation (RGC survival, and nerve fiber layer integrity), and neuroprotective efficacy.

**Methods:**

A systematic literature retrieval from multiple online databases (e.g., PubMed, Web of Science, Embase, Cochrane Library, Scopus, CNKI, WFSD, VIP, and CBM) was conducted through 3 January 2025, to identify relevant animal experimental studies. After the assessment of the risk of bias in the listed articles, this study further computed effect sizes of stem cell transplantation on indices such as IOP, RGC count. Statistical analyses were completed using RevMan 5.3.

**Results:**

In the meta-analysis involving 19 studies, the stem cell transplantation group had significantly lower IOP (MD = -1.55,95% CI = −2.62 to −0.47) at weeks 3 and 4, higher RGC count at weeks 2, 3, and 4 (MD = 23.06,95%CI = 18.22–27.89), and greater nerve fiber layer thickness (MD = 10.69, 95%CI = 9.44–11.94) compared to the control group. Meanwhile, the stem cell transplantation group also had higher retinal BDNF expression at weeks 2 and 4 (MD = 0.75,95%CI = 0.67–0.83), GDNF expression at weeks 1, 2, 3, and 4, and IGF-1 expression (MD = 0.49,95%CI = 0.39–0.58). None of these studies reported any adverse systemic events.

**Conclusion:**

This meta-analysis provides preclinical evidence supporting stem cell transplantation as a multimodal therapeutic strategy for glaucoma, demonstrating significant efficacy in IOP modulation, neurostructural preservation, and neurotrophic factor expression. Given the severity of glaucoma-induced ocular structural damage, it underscores the significance of stem cell transplantation as a secure and promising therapeutic option with notable neuroprotective potential, despite existing translational challenges regarding optimal cell sources, delivery routes, and long-term safety profiles.

**Systematic Review Registration:**

www.inplasy.com, identifier 202520023

## 1 Introduction

Glaucoma is a common pathology of the eye with increased intraocular pressure (IOP) and optic nerve damage as the major manifestations. If left untreated, it can eventually lead to vision loss (Asrani et al., 2024). Millions of people are negatively impacted by glaucoma, second after cataracts in the top list of inducing blindness (Quigley and Broman, 2006; Chen and Shen, 2015). Currently, topical IOP-lowering medications (e.g., prostaglandin analogs, β-blockers), selective laser trabeculoplasty, and filtration surgeries remain the gold-standard therapeutic approaches for glaucoma, demonstrating partial efficacy in delaying disease progression. However, these therapies inherently fail to address the critical pathobiological mechanisms underlying neural repair. Notably, persistent visual field deterioration is reported in approximately 30%–40% of advanced-stage patients, despite their achievement of target IOP levels. This therapeutic gap is further exacerbated by the poor regenerative capacity of adult mammalian retinal ganglion cells (RGCs) and insufficient endogenous production of brain-derived neurotrophic factor (BDNF), glial cell line-derived neurotrophic factor (GDNF), two representative neurotrophic factors indispensable for neuronal survival and axonal regeneration. Emerging preclinical evidence highlights the potential dual therapeutic effects exerted by stem cell-based strategies: modulating IOP through trabecular meshwork remodeling and conferring direct neuroprotection via paracrine factor secretion (e.g., neurotrophins, and growth factors). These biological properties position stem cell therapy as a promising modality to bridge the translational chasm between symptomatic control and true neural restoration in glaucoma management. Therefore, new therapeutic strategies are necessitated to repair the damage to the optic nerve and restore vision (Chen and Ng, 2018).

The emergence of stem cell therapy has offered new hope for glaucoma treatment recently. Stem cells, supported by their self-renewal and differentiation, have shown great promise, particularly in retinal and optic nerve repair. Stem cells have been documented to improve optic nerve damage in glaucoma patients by promoting neuronal regeneration, protecting optic nerve fibers, and reducing IOP (Emre et al., 2023). Therefore, it may be a feasible choice for glaucoma, especially for advanced-stage patients.

Despite the broad prospects, stem cell therapy for glaucoma remains in the experimental stage, primarily evaluated in animal models and preclinical studies. It has not yet been widely applied clinically owing to variations in related technologies/transplantation strategies, ethical-regulatory controversies, uncertainties in risk management, etc. It is expected to realize gradually advanced clinical implementation if these challenges can be addressed. Several animal experiments have shown positive results with stem cells in repairing optic nerves and improving vision (Johnson et al., 2014; Guo et al., 2021). In glaucoma models, certain therapeutic effects have been discovered in various types, including bone marrow-derived mesenchymal stem cells (BMSCs), neural stem cells, and induced pluripotent stem cells. However, several challenges can still not be omitted, such as donor heterogeneity (e.g., age-related mitochondrial dysfunction in BMSCs), immune-mediated adverse reactions, and tumorigenic risks from residual undifferentiated cells, all of which should be further clarified and verified (Kasetty et al., 2022). In response to these limitations, current investigations have focused on innovative strategies involving precision lineage-specific differentiation, immunomodulation (via anti-inflammatory cytokines or hypoxia-mimetic agents to reduce pro-inflammatory cytokine secretion), and delivery system innovation (e.g., biomaterial-encapsulated stem cells), which hold potential to mitigate the occurrence and progression of associated adverse reactions.

By reviewing related animal model studies, this study assessed the efficacy and safety of stem cell transplantation for glaucoma. This study focused on randomized controlled trials (RCTs) using three common glaucoma animal models: laser-induced photocoagulation of the aqueous humor outflow pathway, mechanical optic nerve injury, and laser photocoagulation of episcleral veins to induce ocular hypertension. It aimed to assess their efficacy in controlling IOP, preserving RGC function, and increasing retinal nerve fiber layer thickness. Additionally, the study examined whether treatment enhanced the expression of neurotrophic factors (e.g., BDNF, GDNF, and IGF-I) that promote RGC survival, optic nerve regeneration, and neuronal self-repair, while also evaluating treatment safety. It may provide scientific evidence for stem cell therapy in glaucoma and offer potential reference for its clinical application, thus contributing to the theoretical support for future clinical translation.

## 2 Methods

This study, with corresponding registered in INPLASY (No.202520023), was conducted according to the PRISMA.

### 2.1 Search strategy

Two researchers searched PubMed, Web of Science, Embase, Cochrane Library, Scopus, CNKI, WFSD, VIP, and CBM from their inception to 3 January 2025. The English search terms were: “stem cells”, “stem cell”, “stromal cells”, and “glaucoma”. References from the included studies and related reviews were traced to find out additional references eligible for our analysis.

### 2.2 Eligibility criteria

Inclusion:

(1) Studies involving successfully established and verified animal models of glaucoma.
(2) Studies without restrictions on the induction method of glaucoma.
(3) RCTs.
(4) Studies with no restrictions on animal species, sex, age, or weight.
(5) Studies with the establishment of a placebo or untreated control group.

Exclusion:

(1) Conference abstracts and review articles.
(2) Non-English and Non-Chinese publications.

### 2.3 Data extraction

Screening of relevant literature was completed by two researchers independently. The data were then collated and checked by two other researchers. After removing duplicates, the titles and abstracts were screened to exclude obviously irrelevant studies and review articles. The full texts were then assessed to determine whether these studies should be included. In case of studies with incomplete data, the original authors would be contacted to obtain additional information. Data were calculated from standard errors (SEs), CIs, t or p values for those without standard deviations (SDs), or looked for from the original authors through mail contact. GetData Digitizer 2.20 would be employed to extract data we needed from the available graphs. Studies would be excluded when the above attempts were failed to obtain relevant data. Potential disagreements would be resolved by consulting a third party. Terms of extraction were the title, first author’s name, publication year, animal species, sample size, type of experimental model, stem cell type, administration route, intervention measures, and outcome indicators.

### 2.4 Quality assessment

The SYRCLE’s Risk of Bias tool for animal studies, with 10 recommended items, was used for the assessment. The details related to quality assessment items and corresponding results are summarized in Table 2. The assessment results were classified as “L,” “H,” and “U,” representing low, high, and unclear risk of bias, respectively.

### 2.5 Statistical analysis

RevMan 5.3 was utilized for analyzing the outcome measures of the included studies, with the statistical difference identified by P < 0.05. For continuous outcome measures, when all studies utilized identical animal models and outcome measurement protocols (e.g., consistent IOP measurement techniques or standardized RGC density quantification), data were pooled by the weighted mean difference (WMD) with 95% confidence intervals (CIs). In contrast, the standardized mean difference (SMD) with 95% CIs was applied as the effect size measure to account for methodological heterogeneities in experimental designs or measurement scales across studies. The heterogeneity was tested prior to effect size pooling. A random-effects model was used in the presence of heterogeneity (e.g., I^2^ > 50% and P < 0.1); otherwise, a fixed-effects model was applied. Sensitivity and subgroup analyses were further adopted, in case of significant heterogeneity, by sequentially excluding individual studies to identify the source of heterogeneity. Funnel plot for publication bias was referenced when >9 studies were included for a single outcome measure.

## 3 Results

### 3.1 Literature search

**FIGURE 1:**
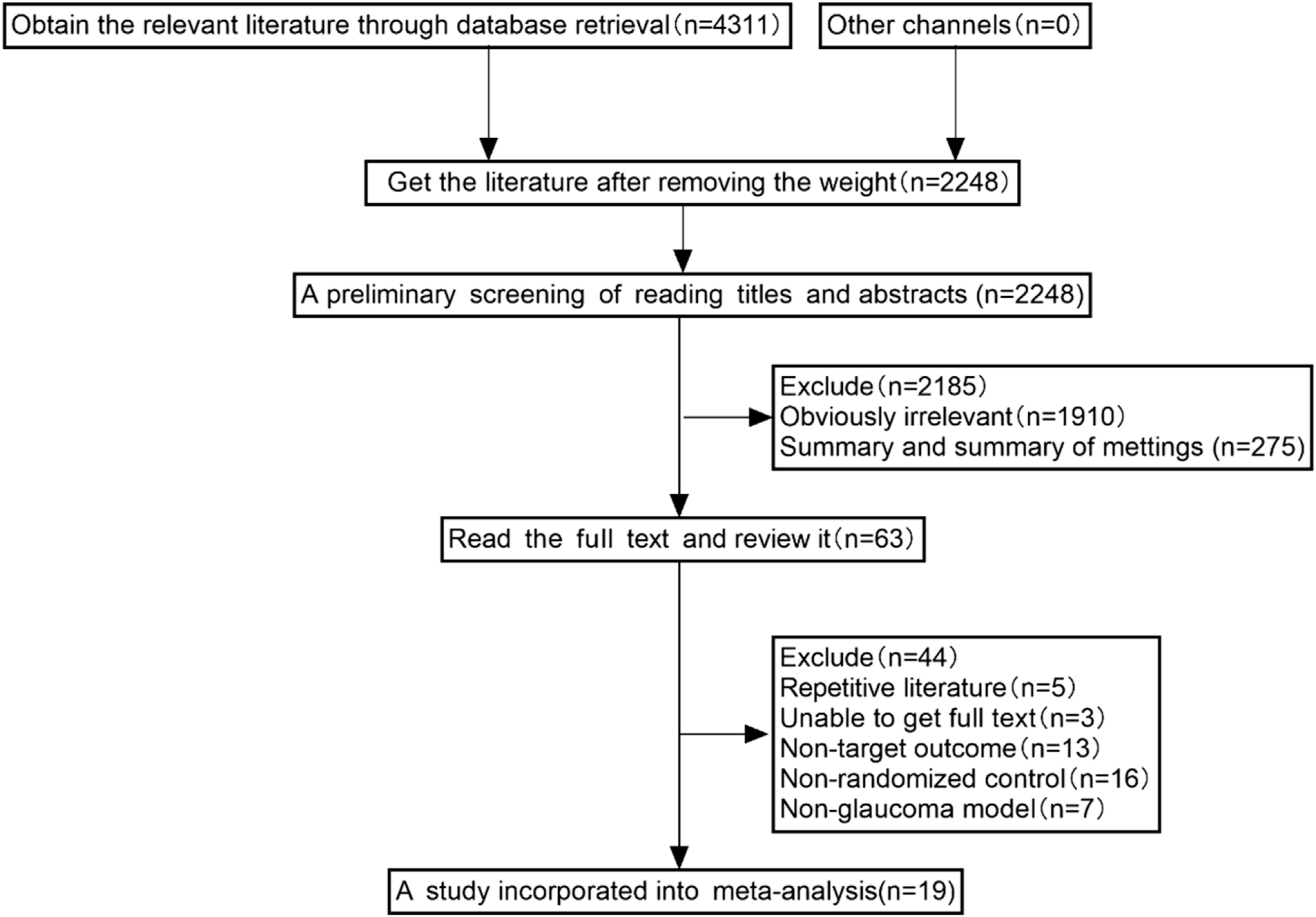
The selection process of studies included for meta-analysis.

Preliminary searching and duplicate removal identified 2,248 articles, including 974 from Embase, 460 from PubMed, 226 from Web of Science, 316 from Scopus, 12 from Cochrane, 112 from CBM, 8 from VIP, 49 from CNKI, and 89 from WFSD. Nineteen studies were included in this meta-analysis after excluding irrelevant studies based on the above inclusion criteria (Figure 1).

### 3.2 Eligible study characterization

As clearly implied in Table 1, this study included 19 RCTs conducted in rats, rabbits, and mice mainly. Six studies were conducted based on a glaucoma animal model using laser photocoagulation of the aqueous humor outflow region, three studies implemented a mechanical optic nerve injury animal model, and three studies created a high IOP animal model utilizing laser photocoagulation of the scleral surface veins. Five studies primarily created a high IOP animal model by injecting physiological saline, sodium hyaluronate, transforming growth factor, Ringer’s solution, or conjunctival fibroblasts into the anterior chamber. One study established a high IOP animal model by subconjunctival injection of dexamethasone, and another study induced a high IOP animal model with prednisone acetate and betamethasone acetate.

**TABLE 1:** Basic features of the eligible studies.

Studies included	Animals	T/C	Modeling	*T*	*C*	Outcome measures	Condition
Guo et al. (2012)	white rabbit	20/20	Subconjunctival injection of dexamethasone	Vitreous cavity injection of NSC	Blank control	⑥	All animals were housed under standard conditions, with 12 h of alternating darkness (7:00–19:00 for the photoperiod). All animal behavior and dosing experiments were completed between 13:30 and 16:00
Sihota et al. (2019)	white rabbit	12/10	Laser photocoagulation of the aqueous humor outflow region	Vitreous cavity injection of hUCBSCs	Blank control	⑥⑦	Animals were housed in pathogen-free conditions and underwent a thorough clinical examination including intraocular pressure evaluation, to exclude any prior ocular or systemic pathology
Liu et al. (2021)	C57BL/6 Mice	14/14	Injection of physiological saline into the anterior chamber	Vitreous cavity injection of hUCMSCs	Equal volume of saline solution	②	Experimental animals in a room with the adjustable climate where the light/dark cycle ratio is 12 h:12 h. Food and water were available to the animals
Zhao et al. (2011)	SD Rats	45/45	Mechanical optic nerve injury model	Vitreous cavity injection of hUCBSCs	Equal volume of saline solution	②③④⑦	—
Evangelho et al. (2024)	white rabbit	10/5	Induction with prednisone acetate and betamethasone acetate	Vitreous cavity injection of hWJ-MSCs	Blank control	①	The animals were housed in a controlled environment with 50% ± 20% relative humidity, room temperature of 15ºC-23°C and 12-h light/12-h darkness cycle
Emre et al. (2015)	Wistar Rats	32/8	Anterior chamber injection of sodium hyaluronate	Vitreous cavity injection of BMSCs or ATMSCS	Equal volume of PBS	①②⑥⑦	The environmental temperatures were kept constant at 19°C–23°C, and the humidity was controlled. The rooms were lit with fluorescent lights on a 12-h on-off light cycle (06:00 a.m.-06:00 p.m.)
Li et al. (2018)	SD Rats	30/30	Mechanical optic nerve injury model	Vitreous cavity injection of ASCs	Equal volume of PBS	①	All animals were raised in a specific-pathogen-free animal house with a room temperature of 22°C–24°C, 12-h light-dark cycle, and a relative humidity of 50%–60% —
Gu et al. (2016)	SD Rats	15/15	Laser photocoagulation of the scleral surface veins	Vitreous cavity injection of RSC	Equal volume of PBS	①③⑤	
Zhou (2013)	SD Rats	6/6	Laser photocoagulation of the corneal limbal vascular network and three superficial scleral veins	Vitreous cavity injection of RSC	Equal volume of PBS	①⑤⑥	—
Yu et al. (2023)	SD Rats	20/20	Anterior chamber injection of conjunctival fibroblasts	Vitreous cavity injection of MSCs	Equal volume of PBS	①	Animals were maintained on a 12-h light-dark cycle and were dark-adapted for at least 2 h before the experiments
Manuguerra-Gagné et al. (2013)	Brown Norway Rats	12/12	Laser photocoagulation of the aqueous humor outflow region	Anterior chamber injection of MSCs	Equal volume of saline solution	①⑥	—
Mead et al. (2016)	SD Rats	12/6	Anterior chamber injection of transforming growth factor	Vitreous cavity injection of DPSC、ADSC、BMSCs	Blank control	①	Animals housed in conditions of 21°C and 55% humidity under a 12 h light/dark cycle with a daytime luminance of 80 lux, given food and water
Roubeix et al. (2015)	Long Evans Rats	25/25	Laser photocoagulation of three superficial scleral veins	Anterior chamber injection of BMSCs	Equal volume of culture medium	①②	Animals were kept in pathogen-free conditions with food and water available *ad libitum* and were housed in a 12-h light/12-h dark cycle
Hu et al. (2013)	SD Rats	14/7	Laser photocoagulation of the aqueous humor outflow region	Vitreous cavity injection of BMSCs	Blank control	①	Animals maintained on a 12/12 dark/light cycle with free access to food and water in single housing
Tian et al. (2020)	Lewis Rats	20/10	Laser photocoagulation of the aqueous humor outflow region	Vitreous cavity injection of BMSCs	Equal volume of PBS	①②③	Lewis rats are housed in standard plastic cages with sawdust litter at room temperatures of 21°C ± 2°C, animals have unrestricted access to food and water, maintain a 12 h light/12 h dark cycle, and acclimatize the rats to the environment for at least 2 weeks prior to the experiment
Johnson et al. (2014)	SD Rats	30/15	Laser photocoagulation of the aqueous humor outflow region	Vitreous cavity injection of PDGF – AA or PDGF – AB	Equal volume of PBS	①	—
Bull et al. (2009)	Lewis Rats	10/8	Laser photocoagulation of the aqueous humor outflow region	Vitreous cavity injection of OPCs	Equal volume of PBS	⑥	Animals had unrestricted access to food and water, and were maintained on a 12-h light/dark cycle
Liu (2006)	SD Rats	42/42	Mechanical optic nerve injury model	Subvitreal injection of NSC	Equal volume of PBS	①③④	—
Wang (2004)	Wistar Rats	12/12	Anterior chamber injection of Ringer’s solution	Subretinal injection of BMSCs	Equal volume of PBS	⑥⑦	—

T, experimental group; C, control group; OPCs, Oligodendrocyte precursor cells; BMSCs, Bone marrow mesenchymal stem cells; ATMSCs, Adipose-derived mesenchymal stem cells; hUCBSCs, Human umbilical cord blood stem cells; RSC, retinal stem cells; NSC, neural stem cells; hUC MSCs, Human umbilical cord mesenchymal stem cells; hWJ-MSCs, Human Wharton’s jelly mesenchymal stem cells; ASCs, Adipose-derived stem cells; MSCs, Mesenchymal stem cells; DPSC, dental pulp stem cells; ADSC, Adipose-derived stem cells; PDGF, Platelet-derived growth factor; ① IOP; ② RGC, count; ③ Expression of retinal BDNF; ④ Expression of retinal GDNF; ⑤ Expression of retinal IGF-I; ⑥ Survival, migration, differentiation, and integration of transplanted stem cells; ⑦ Adverse reactions; and ⑧ Retinal nerve fiber layer thickness.

### 3.3 Quality assessment

**FIGURE 2:**
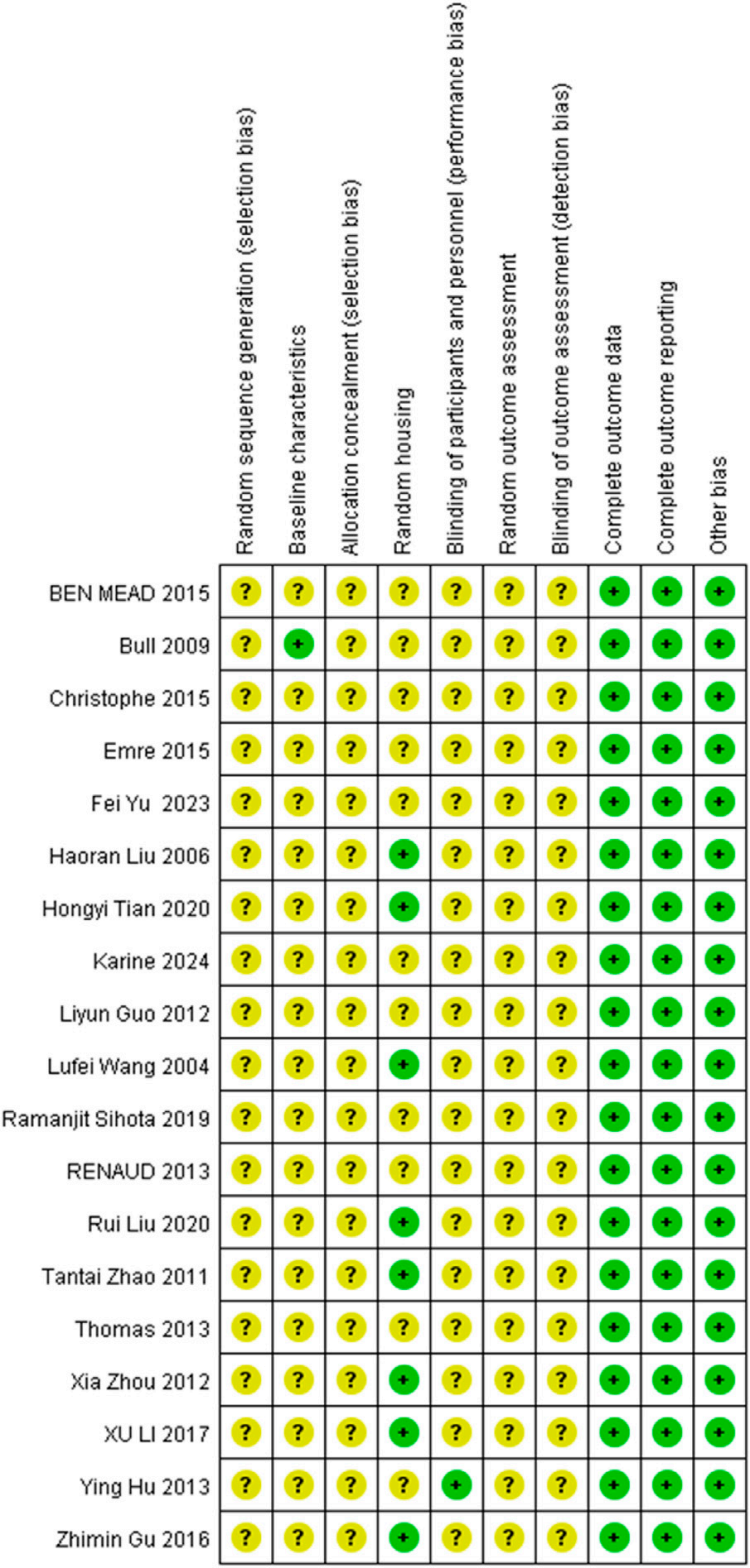
Review of authors’ judgement about each risk of bias item for each study included. Rows correspond to individual studies, while columns represent the assessment items. A question mark (?) denotes an unclear risk of bias, while a plus sign (+) indicates a low risk of bias.

**FIGURE 3:**
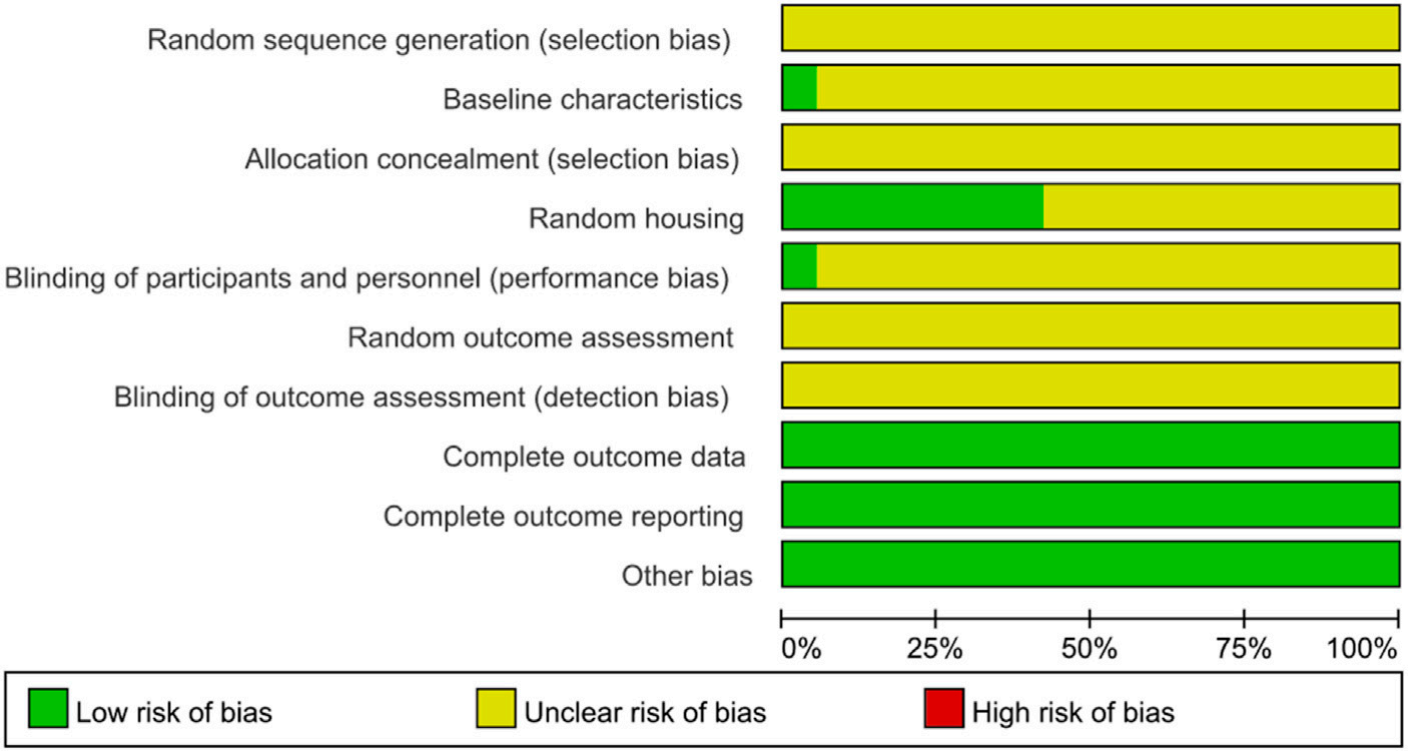
Review of authors’ judgement about each risk of bias item presented as percentages across all included studies. Rows represent different assessment categories, with the length of color-coded bar segments in each row corresponding to their respective percentages.

All the 19 studies showed relatively low attention to bias concerns. There were no sufficiently clear descriptions of key methodological aspects, such as random allocation and concealment of allocation. The experimenters were inevitably aware of whether the animals received stem cell transplantation, and in the absence of clear statements on whether the subjects were randomly assigned to treatment groups, all of which might have contributed to certain biases. Nevertheless, all studies included here provided relatively detailed reports of their research processes. Therefore, their methodological quality could be considered reliable and acceptable (Table 2; Figures 2, 3).

**TABLE 2:** Assessment of bias risk in the included studies.

Studies included	①	②	③	④	⑤	⑥	⑦	⑧	⑨	⑩
Guo et al. (2012)	U	U	U	U	U	U	U	L	L	L
Sihota et al. (2019)	U	U	U	U	U	U	U	L	L	L
Liu et al. (2021)	U	U	U	L	U	U	U	L	L	L
Zhao et al. (2011)	U	U	U	L	U	U	U	L	L	L
Evangelho et al. (2024)	U	U	U	U	U	U	U	L	L	L
Emre et al. (2015)	U	U	U	U	U	U	U	L	L	L
Li et al. (2018)	U	U	U	L	U	U	U	L	L	L
Gu et al. (2016)	U	U	U	L	U	U	U	L	L	L
Zhou (2013)	U	U	U	L	U	U	U	L	L	L
Yu et al. (2023)	U	U	U	U	U	U	U	L	L	L
Manuguerra-Gagné et al. (2013)	U	U	U	U	U	U	U	L	L	L
Mead et al. (2016)	U	U	U	U	U	U	U	L	L	L
Roubeix et al. (2015)	U	U	U	U	U	U	U	L	L	L
Hu et al. (2013)	U	U	U	U	L	U	U	L	L	L
Tian et al. (2020)	U	U	U	L	U	U	U	L	L	L
Johnson et al. (2014)	U	U	U	U	U	U	U	L	L	L
Bull et al. (2009)	U	L	U	U	U	U	U	L	L	L
Liu (2006)	U	U	U	L	U	U	U	L	L	L
Wang (2004)	U	U	U	L	U	U	U	L	L	L

①Random sequencing; ②Baseline characteristics; ③Allocation concealment; ④Random housing; ⑤Blinding of participants and personnel; ⑥Random outcome assessment; ⑦Blinding of outcome assessment; ⑧Complete outcomes; ⑨Complete outcome reporting; ⑩Other bias. H, high risk of bias; L, low risk of bias; U, uncertain.

### 3.4 Effect on IOP

**FIGURE 4:**
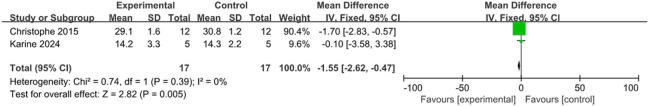
The forest plot showed a decrease in intraocular pressure in the stem cell transplant group at the third week.

**FIGURE 5:**
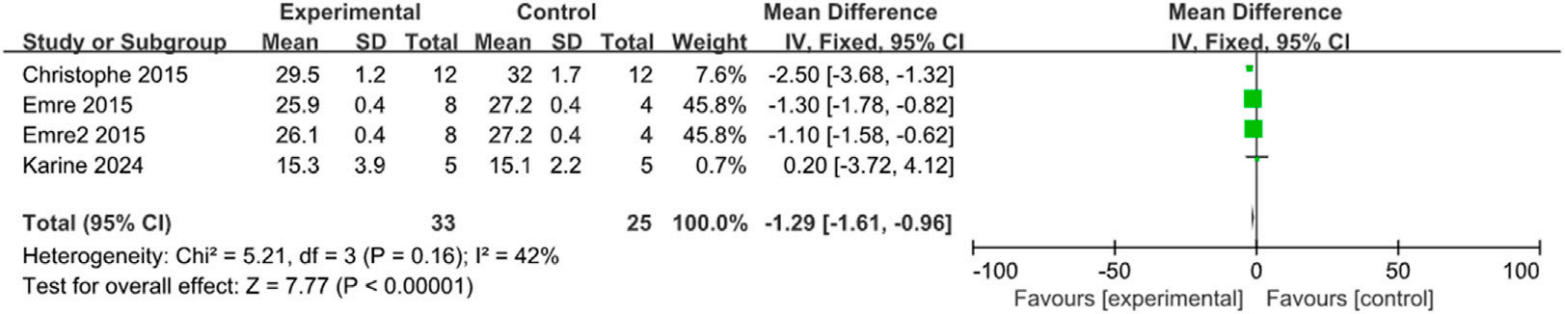
The forest plot showed a decrease in intraocular pressure in the stem cell transplant group at the fourth week.

The control of IOP is a crucial parameter during glaucoma treatment. With transplantation and control groups established, two studies measured the changes in IOP at the third week, and three studies at the fourth week, involving 72 rats and 15 rabbits. According to the meta-analysis inter-group comparison revealed a statistically significant difference at both the third week [MD = −1.55, 95% CI (−2.62, −0.47), P = 0.005, I^2^ = 0%, Figure 4, Supplementary Figure S1], and the fourth week (MD = −1.29, 95% CI (−1.61, −0.96), P < 0.001, I^2^ = 42%, Figure 5, Supplementary Figure S2). Therefore, stem cell transplantation resulted in lower IOP at both the third and fourth weeks.

### 3.5 Effect on RGC count

**FIGURE 6:**
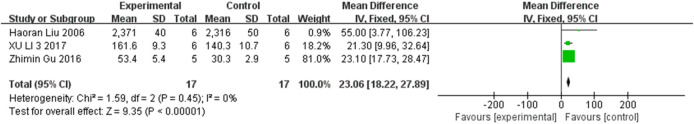
The forest plot shows an increase in RGC count in the stem cell transplant group at the second week.

**FIGURE 7:**
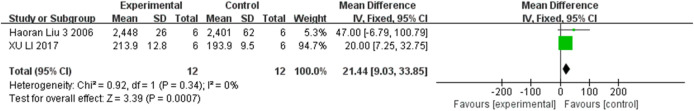
The forest plot shows an increase in RGC count in the stem cell transplant group at the third week.

**FIGURE 8:**
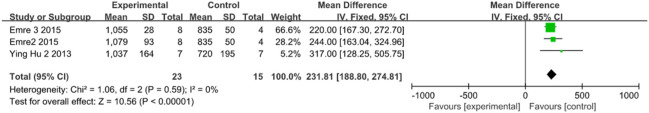
The forest plot shows an increase in RGC count in the stem cell transplant group at the fourth week.

The progressive loss of RGCs is also a critical component of the pathogenesis of glaucoma. Three studies, two studies, and two studies respectively measured the changes in RGC count at the second, third, and fourth weeks in the two groups, involving 96 rats. Consequently, inter-group comparison showed statistically significant differences at the second week (MD = 23.06, 95% CI (18.22, 27.89), P < 0.001, I^2^ = 0%, Figure 6, Supplementary Figure S3), the third week (MD = 21.44, 95% CI (9.03, 33.85), P = 0.001, I^2^ = 0%, Figure 7, Supplementary Figure S4), and the fourth week (MD = 231.81, 95% CI (188.80, 274.81), P < 0.001, I^2^ = 0%, Figure 8, Supplementary Figure S5). Collectively, stem cell transplantation would elevate RGC count at the second, third, and fourth weeks.

### 3.6 Effect on retinal BDNF expression

**FIGURE 9:**
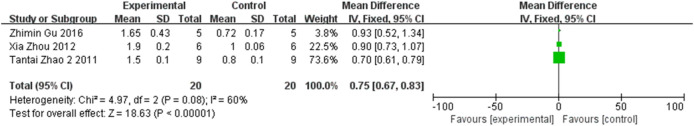
The forest plot showed that retinal BDNF expression was upregulated in the stem cell transplant group at the second week.

**FIGURE 10:**
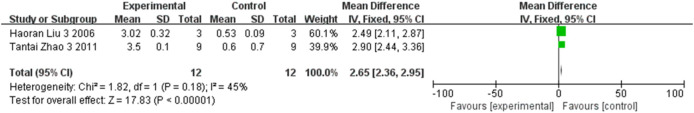
The forest plot showed that retinal BDNF expression was upregulated in the stem cell transplant group at the fourth week.

Three studies and two studies respectively compared the expression of retinal BDNF at the second and fourth weeks between groups, involving a total of 64 rats. Meta-analysis results showed, statistically significant inter-group differences at the second week (MD = 0.75, 95% CI (0.67, 0.83), P < 0.001, I^2^ = 60%, Figure 9, Supplementary Figure S6), and the fourth week (MD = 2.65, 95% CI (2.36, 2.95), P < 0.001, I^2^ = 45%, Figure 10, Supplementary Figure S7). Based on these findings, stem cell transplantation would upregulate the retinal BDNF expression at both the second and fourth weeks.

### 3.7 Effect on retinal GDNF expression

**FIGURE 11:**
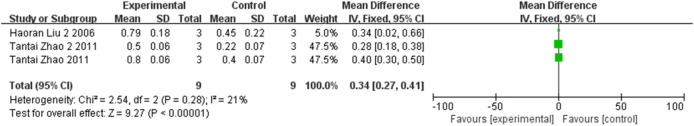
The forest plot shows that retinal GDNF expression was up-regulated in the stem cell transplant group at the first week.

**FIGURE 12:**
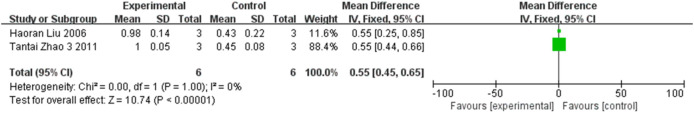
The forest plot shows that retinal GDNF expression was up-regulated in the stem cell transplant group at the second week.

**FIGURE 13:**
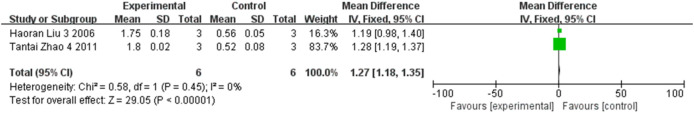
The forest plot shows that retinal GDNF expression was up-regulated in the stem cell transplant group at the third week.

**FIGURE 14:**
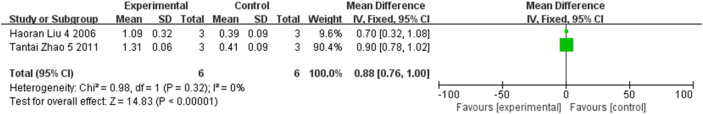
The forest plot shows that retinal GDNF expression was up-regulated in the stem cell transplant group at the fourth week.

The expression of retinal GDNF at the first, second, third, and fourth weeks were compared respectively in three studies, two studies, two studies, and two studies, involving a total of 54 rats. As indicated by the meta-analysis, statistically significant differences between groups were observed at the first week (MD = 0.34, 95% CI (0.27, 0.41), P < 0.001, I^2^ = 21%, Figure 11, Supplementary Figure S8), the second week (MD = 0.55, 95% CI (0.45, 0.65), P < 0.001, I^2^ = 0%, Figure 12, Supplementary Figure S9), the third week (MD = 1.27, 95% CI (1.18, 1.35), P < 0.001, I^2^ = 0%, Figure 13, Supplementary Figure S10), and the fourth week (MD = 0.88, 95% CI (0.76, 1.00), P < 0.001, I^2^ = 0%, Figure 14, Supplementary Figure S11). All together, stem cell transplantation would elevate the retinal GDNF expression at the first, second, third, and fourth weeks.

### 3.8 Effect on nerve fiber layer thickness

**FIGURE 15:**
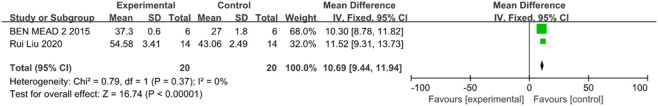
The forest plot showed that the thickness of the nerve fiber layer in the stem cell transplantation group was higher than that in the control group.

Two studies compared the nerve fiber layer thickness between groups, involving 12 rats and 24 rabbits. Meta-analysis results showed a statistically significant difference between groups (MD = 10.69, 95% CI (9.44, 11.94), P < 0.001, I^2^ = 0%, Figure 15, Supplementary Figure S12), supporting higher nerve fiber layer thickness in the stem cell transplantation group.

### 3.9 Effect on retinal IGF-1 expression

**FIGURE 16:**
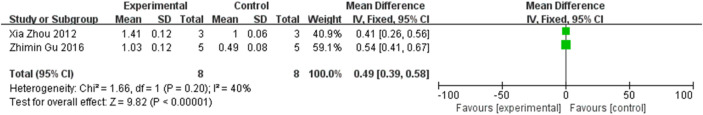
The forest plot shows an increase in retinal IGF-1 expression in the stem cell transplantation group.

Two studies compared inter-group difference in the expression of retinal IGF-1, involving 16 rats. A statistically significant difference was found between groups (MD = 0.49, 95% CI (0.39, 0.58), P < 0.001, I^2^ = 40%, Figure 16, Supplementary Figure S13). It can be concluded that stem cell transplantation would increase retinal IGF-1 expression.

### 3.10 Stem cell migration, differentiation, and integration

For further investigation, we focused on 7 relevant studies, involving 94 rats and 62 rabbits, all of which performed descriptive analysis of the results. Guo Liyun et al. reported a 5-month-survival of transplanted cells attached to the lens after transplantation of neural stem cells (NSCs) into the vitreous body of the rabbit eye. The results of R’s study showed that stem cell injection retained the trabecular bundle structure at 4 weeks post-operation compared to the processing of without stem cell injection, with the trabecular gap widening; and the transplanted stem cells survived in the trabecular tissue for 4–12 weeks. Emre et al. found that, at 2 weeks post-transplantation, adipose-derived mesenchymal stem cells (ATMSCs) and BMSCs integrated into the ganglion cell layer and inner nuclear layer of the host retina; and at 4 weeks, ATMSCs were integrated into the ganglion cell layer. Wang Lufei et al. demonstrated that the transplanted cells survived for at least 30 days in the subretinal space and integrated with the host retinal structure when implementing subretinal transplantation of BMSCs. Further analysis showed that these BMSCs expressed the neuronal cell-specific marker protein P55, suggesting their potential differentiation into neuronal cells. In another study on migration and differentiation behaviors of retinal stem cells (RSCs) by Zhou Xia et al., RSCs were distributed throughout the retinal surface by 14 days after vitreous transplantation, with a higher density near the injection site. Confocal microscopy observations indicated that RSCs fused into the host retina’s nerve fiber layer and ganglion cell layer. Manuguerra-Gagné et al. found that transplanted mesenchymal stem cells (MSCs) showed short-term survival in the anterior chamber and exhibited specific migratory abilities. At the laser injury site, a large number of labeled MSCs were detected 24 h post-transplantation, while the cell count in the intact region was only half of that in the laser injury area. At 96 h post-transplantation, MSCs were difficult to detect in the anterior chamber, suggesting their possible clearance by host ocular macrophages. In addition, Bull et al. reported that oligodendrocyte precursor cells (OPCs) transplanted into the vitreous survived for 4 weeks, but only a small number of cells differentiated into myelinated oligodendrocytes expressing myelin basic protein, while a few OPCs initiated differentiation into neurons and astrocytes within the eye.

### 3.11 Adverse reactions

Four studies involving 154 rats and 22 rabbits were conducted to observe the presence of any adverse reactions. All four studies performed descriptive analysis of the results. The study by Ramanjit et al. showed that in rabbits with a glaucoma model induced by laser photocoagulation of the aqueous humor outflow region, no evidence of inflammation, IOP changes (±2 mmHg or greater), or lens opacification was observed in any of the eyes injected with human umbilical cord blood stem cells (hUCBSCs). Tantai et al. found no abnormal appearances or rejection responses in the rats during the first, second, third or fourth week post-transplantation in a glaucoma model created by mechanical optic nerve injury, without death of all rats. Emre et al. reported no intraocular tumor formation at 2 and 4 weeks post-transplantation after stem cell transplantation in a glaucoma model established by anterior chamber injection of sodium hyaluronate. Wang Lufei et al. concluded no rejection or intraocular infection occurred at 10, 20, or 30 days after stem cell transplantation in a model of ischemia-reperfusion created by anterior chamber injection of Ringer’s solution.

## 4 Discussion

Glaucoma is characterized by the progressive degeneration of RGCs, which has been accepted to be one major cause of irreversible blindness worldwide. Factors such as oxidative stress and neuroinflammation play critical roles in the pathogenesis of glaucoma (Vohra et al., 2013; Fan et al., 2021). Currently, glaucoma is generally treated by lowering IOP, but many patients, even with effective IOP control, still experience irreversible optic nerve damage and vision loss. Therefore, it is of paramount importance for implementing effective measures to protect RGCs, particularly for the preservation of optic nerve function. Stem cells have recently gained widespread attention due to their high regenerative and differentiation potential (Dahlmann-Noor et al., 2010; Lin et al., 2019). So far, effective treatment strategies have not been widely applied, despite significant research progress. Existing studies were conducted based on the establishment of specific animal models, mainly exploring the effects of stem cells on IOP regulation (Son et al., 2023). There is insufficient data on the comprehensive role in optic nerve repair, neuroprotection, and functional recovery. Therefore, to solid foundation for future clinical use, it is pivotal for evaluating the efficacy and safety of this treatment in related trials to advance the clinical application potential of stem cell therapy in glaucoma treatment (Wang et al., 2024).

This study included 19 RCTs in three common animal models (e.g., rats, rabbits, and mice) of glaucoma to confirm the efficacy and safety of stem cell therapy systematically. In terms of efficacy, stem cell transplantation was effective in controlling IOP and improving RGC function. It could increase the expression of BDNF, GDNF, and IGF-1 in the retina, and improve retinal nerve fiber layer thickness to some extent. For further elucidation of the biological implications of stem cell transplantation, this study demonstrated that cell type and delivery route critically regulated survival duration, spatial localization, and differentiation potential (e.g., stem cells can differentiate into neuronal cells, myelinated oligodendrocytes, and astrocytes). Transplanted stem cells exhibited therapeutic capabilities through the expression of neuronal markers, chemotactic migration toward injury sites, retinal structural integration, and modulation of pathological microenvironments. In addition, no adverse reaction was reported in all studies included in this meta-analysis, further confirming its safety for glaucoma treatment. These findings challenge the previously conservative views on the safety of stem cell therapy, holding significant clinical application value.

Compared to most studies (Emre et al., 2023; Zhou et al., 2013; Ching et al., 2019; Chen et al., 2022), this research validated the efficacy of stem cell transplantation across multiple animal models, which can control IOP, repair retinal nerve damage, and enhance optic nerve function. Moreover, this study provides further evidence that this therapy can contribute to optic nerve protection and regeneration by promoting neurotrophic factor expression and increasing retinal nerve fiber layer thickness, in addition to effectively regulating IOP. Finally, we acquire more reliable evidence for the safety of stem cell therapy in glaucoma treatment. Collectively, this study provides important references for future research in this field and lays the foundation for clinical translation of stem cell therapy.

Previous studies have investigated the protective effects and safety of stem cell transplantation on optic nerve changes in rat glaucoma models (Liu et al., 2019). However, these studies primarily focused on rat models and presented relatively outdated data. In contrast, the present study offers significant innovation and advancement in several aspects. First, this study incorporates the latest experimental data, with notable improvements in both the quantity and quality of the data, covering a range of animal models including rats, mice, and rabbits. This broadens the generalizability and extrapolation of the findings. Additionally, this research provides a more comprehensive exploration of the effects of stem cell transplantation in the treatment of glaucoma, extending beyond the changes in the optic nerve to a broader perspective. Therefore, the present study expands on previous research in terms of both the choice of animal models and the scope of investigation, offering more extensive data to support the safety and efficacy of stem cell therapy for glaucoma.

Even so, there are still certain limitations in this meta-analysis. Firstly, there were relatively limited animal models, and since these models differ from human ocular structures and disease mechanisms, which might compromise stem cell treatment outcomes. Secondly, heterogeneous results might generate owing to differences in the type, dosage, and method of stem cell therapy. Additionally, there might be risks of selection bias, implementation bias, and subjective outcome measure bias due to variations in the quality of relevant literature and experimental methods when selecting eligible studies for meta-analysis. Lastly, due to inadequate monitoring of the long-term effect of stem cell transplantation, there might be a lack of comprehensive evaluation of potential pathogenic risks. Future studies should consider using a wider range of animal models, particularly primates, to better decipher human pathological characteristics. Moreover, standardized protocols should be established for the selection of stem cell types and dosages to reduce heterogeneity. It is crucial to improve literature selection criteria and quality control, while addressing possible long-term risks of this therapy.

## 5 Conclusion

Stem cell transplantation shows potential benefits in the treatment of glaucoma, with promising safety and efficacy. However, sufficient long-term, systematic data is required for further validation due to the limited number and average quality of current studies. Therefore, future large-scale, high-quality experimental studies should be conducted to further validate these findings and provide more robust scientific evidence.

## Data Availability

The original contributions presented in the study are included in the article/Supplementary Material, further inquiries can be directed to the corresponding authors.
