# Supplementary figures and images for: Efficacy and safety of stem cells in the treatment of glaucoma: systematic review and meta-analysis based on animal experiments
Source: Front Pharmacol. 2025 Jul 1;16:1587440. doi: 10.3389/fphar.2025.1587440 (PMC12259681; doi:10.3389/fphar.2025.1587440)

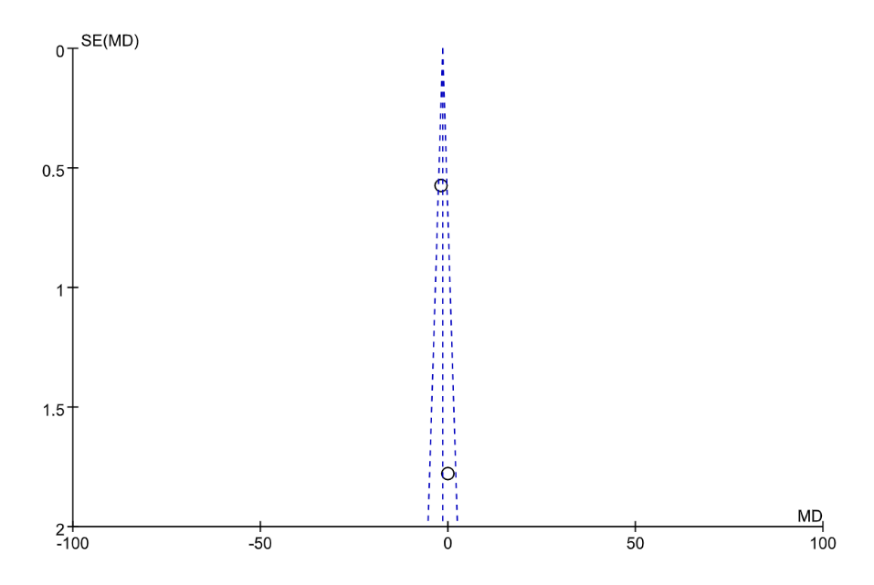


Fig.S1


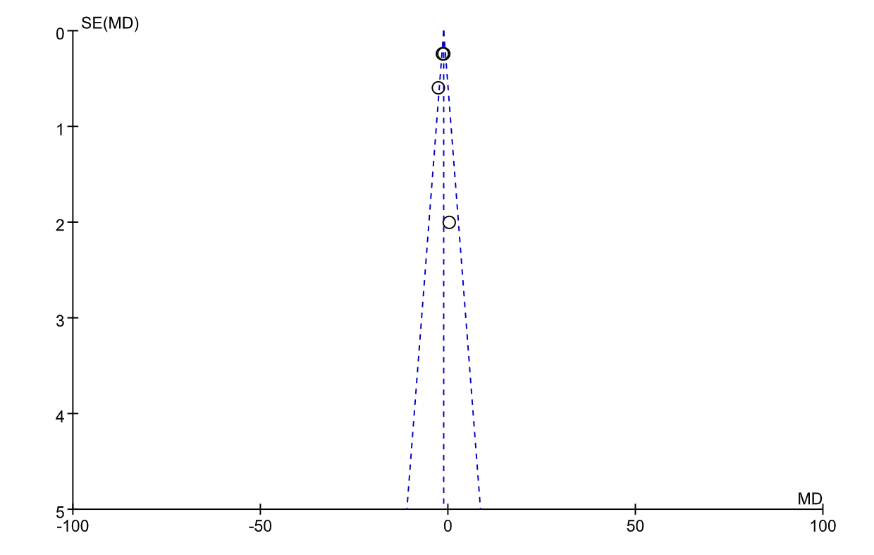


Fig.S2


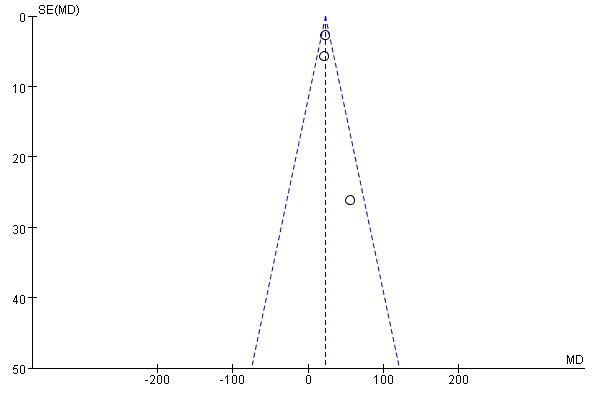


Fig.S3


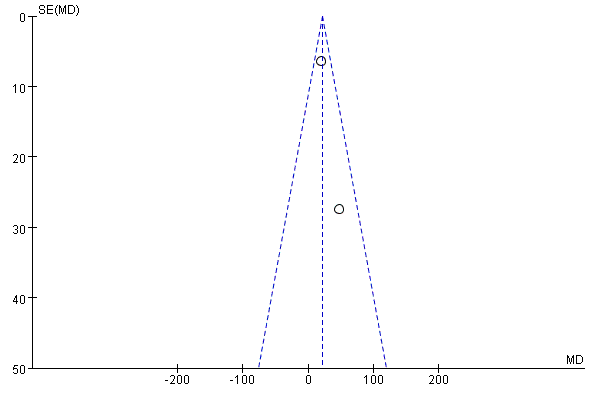


Fig.S4


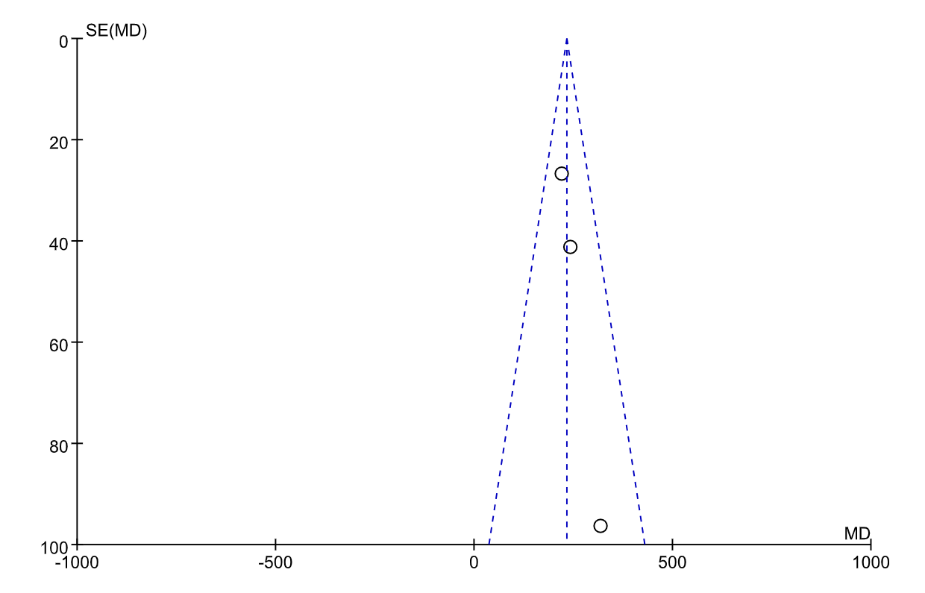


Fig.S5


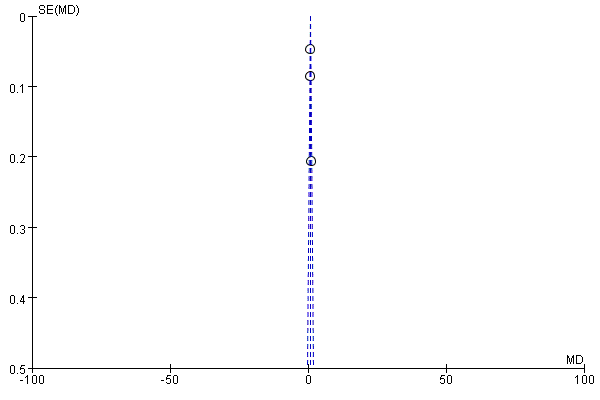


Fig.S6


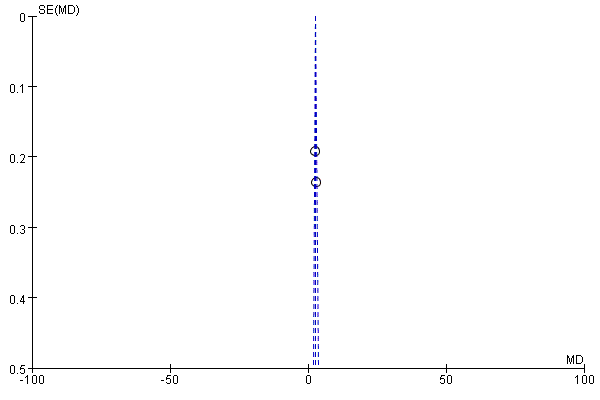


Fig.S7


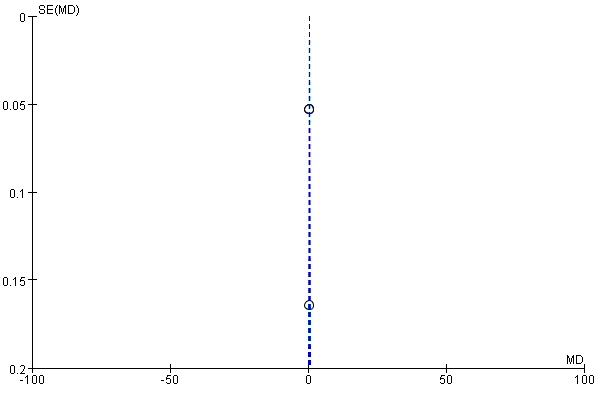


Fig.S8


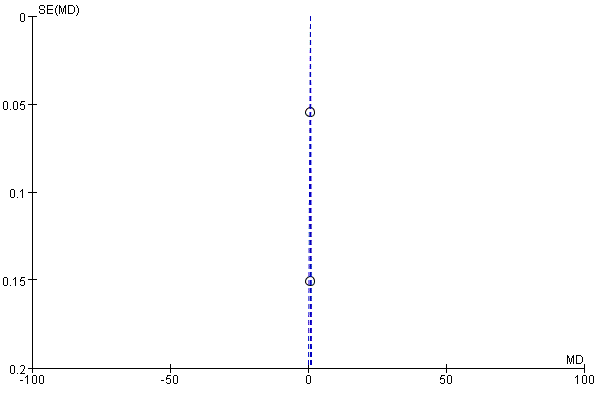


Fig.S9


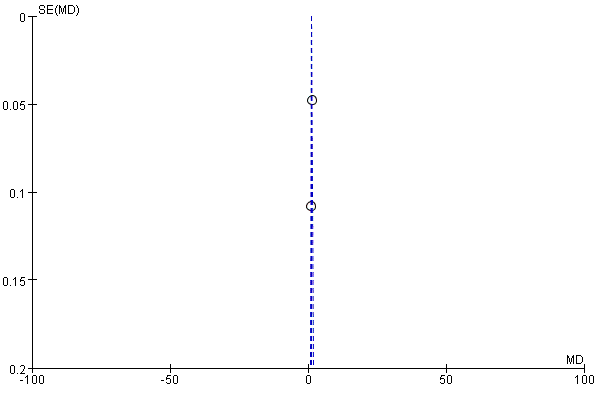


Fig.S10


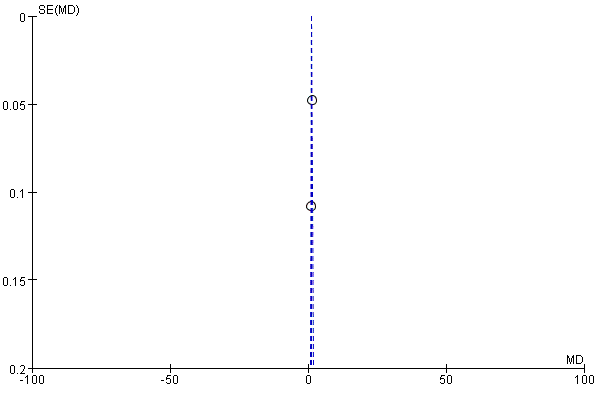


Fig.S11


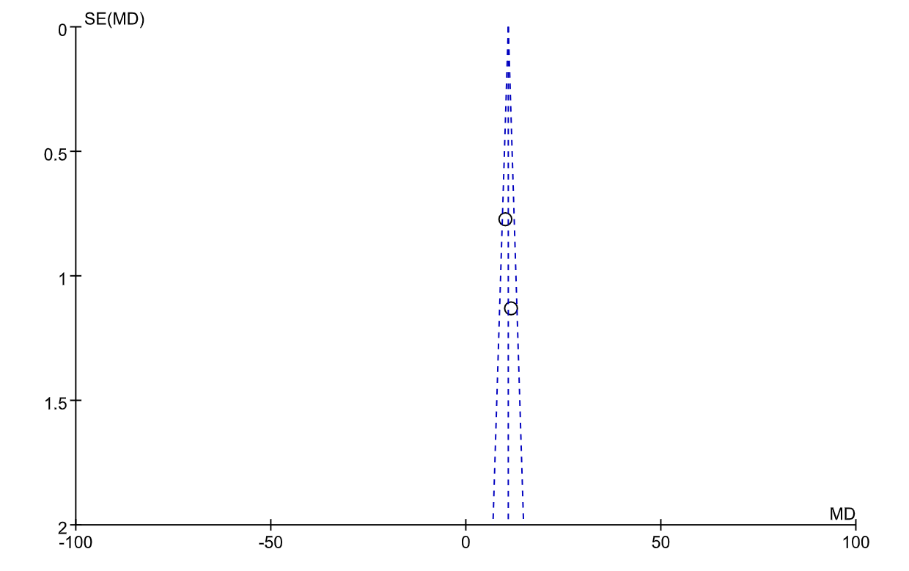


Fig.S12


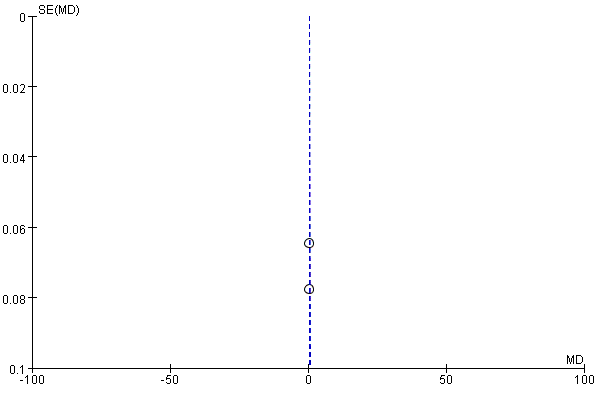


Fig.S13

Supplement: Supplementary file 1 [file DataSheet1.docx]
